# Ischemia‐reperfusion injury and hypoglycemia risk in insulin‐treated T1DM rats following different modalities of regular exercise

**DOI:** 10.14814/phy2.12201

**Published:** 2014-11-20

**Authors:** Matthew W. McDonald, Katharine E. Hall, Mao Jiang, Earl G. Noble, C.W. James Melling

**Affiliations:** 1 School of Kinesiology, Faculty of Health Sciences, University of Western Ontario, London, Ontario, Canada; 2 Health and Rehabilitation Sciences, Faculty of Health Sciences, University of Western Ontario, London, Ontario, Canada; 3 Lawson Health Research Institute, University of Western Ontario, London, Ontario, Canada

**Keywords:** Blood glucose, cardiovascular disease, Hsp70, streptozotocin, type 1 diabetes mellitus

## Abstract

While regular exercise is known to improve cardiovascular function, individuals with type 1 diabetes mellitus (T1DM) have an increased risk for exercise‐induced hypoglycemia. Clinical data suggest that higher intensities of acute exercise may alleviate the onset of hypoglycemia; however, the cardiovascular benefit from these forms of exercise in patients with T1DM has yet to be established. The purpose of this study was to investigate the cardiovascular benefit of different regular exercise regimes, while monitoring blood glucose concentrations during the post‐exercise period. Fifty rats (8‐week‐old Sprague–Dawley male) were equally divided into the following groups: nondiabetic sedentary (C), diabetic sedentary (DS), diabetic low‐intensity aerobic exercise (DL), diabetic high‐intensity aerobic exercise (DH) or diabetic resistance exercise (DR). Diabetes was induced using multiple streptozotocin injections (5×; 20 mg/kg) while subcutaneous insulin pellets maintained glycemia in a range typical for individuals that exercise with T1DM. Exercise consisted of six weeks of treadmill running (DL and DH) or weighted ladder climbs (DR). The cardiovascular benefit of each exercise program was determined by the myocardial recovery from ischemia‐reperfusion injury. Exercise‐related cardiovascular protection was dependent on the exercise modality, whereby DH demonstrated the greatest protection following an ischemic‐reperfusion injury. Each exercise modality caused a significant decline in blood glucose in the post‐exercise period; however, blood glucose levels did not reach hypoglycemic concentrations (<3.0 mmol/L) throughout the exercise intervention. These results suggest that elevating blood glucose concentrations prior to exercise allows patients with T1DM to perform exercise that is beneficial to the myocardium without the accompanying risk of hypoglycemia.

## Abstract (precis)

e12201

This manuscript examined the impact of different exercise modalities on cardiovascular health in type 1 diabetic rats. The manuscript also accounted for the risk of exercise‐induced hypoglycemia associated with each exercise modality, as hypoglycemia is still considered the major barrier to participating in regular activity for many patients with type 1 diabetes mellitus.

## Introduction

Ischemic heart disease is a major complication that is associated with significant morbidity and mortality, particularly for patients with type 1 diabetes mellitus (T1DM) (Orchard et al. 2006). Experimental evidence supports the view that populations with T1DM are more susceptible to ischemia, as hearts of streptozotocin (STZ)‐induced diabetic rats are more sensitive to damage following ischemic‐reperfusion injury (I/R‐injury), as demonstrated by increased infarct size (Forrat et al. 1993). Regular exercise can improve cardiovascular function, as well as other diabetes‐related complications including bone health, body composition, kidney function, insulin sensitivity, and overall quality of life (Daul et al. 2004; Knap et al. 2005; Imayama et al. 2011; Chimen et al. 2012; Melling et al. 2013). Despite these benefits, many patients with T1DM refrain from participating in regular exercise, largely due to the increased risk of experiencing hypoglycemia. Exercise is the most frequently identified cause of known hypoglycemia, while fear of experiencing a hypoglycemic event is the number one barrier for exercise participation for patients with diabetes (Bhatia and Wolfsdorf 1991; Brazeau et al. 2008).

The Canadian Diabetes Association guidelines advise patients with diabetes to participate in regular exercise of moderate intensity (50–70% maximal heart rate [MHR]) to vigorous intensity (>70% MHR), at least three times a week for a total of 150 min (Canadian Diabetes Association Clinical Practice Guidelines Expert Committee 2008; Armstrong and Sigal 2013). However, there is a lack of evidence that regular exercise is capable of improving the primary clinical treatment outcome measure, HbA1c, which has led many to question whether exercise is as advantageous to patients with T1DM as it is for those with Type 2 diabetes mellitus (Chimen et al. 2012; Makura et al. 2013). In fact, patients with T1DM regularly compensate their glucose management by elevating pre‐exercise glucose concentrations through a reduction in insulin dosage to offset the risk of hypoglycemia (Tsalikian et al. 2006). In a recent study, we examined the role of a ten week moderate intensity aerobic exercise training program on cardiac function in poorly controlled STZ‐induced T1DM rats. (Melling et al. 2013). Maintaining T1DM rats in a moderately hyperglycemic range through insulin supplementation was conducted to measure the cardiovascular benefit of regular aerobic exercise in a T1DM model which more accurately reflects those individuals with T1DM who exercise (Tsalikian et al. 2006; Younk et al. 2011; Melling et al. 2013). Moderate intensity regular exercise led to significant improvements in cardiovascular function, as evidenced by an improved ratio of the Early (E) and late (A) ventricular filling velocities (E/A ratio: a marker of left ventricle diastolic function), as well as improvements in other diabetic complications including bone density, insulin sensitivity measures, and body composition (Melling et al. 2013). In rats with T1DM, regular exercise also led to a significant increase in Hsp70 protein content in the heart to the same degree as nondiabetic exercised rats (Melling et al. 2013). Elevated Hsp70 is believed to combat diabetes‐related damage in many organ systems through enhanced oxidant defenses, which provides a potential mechanism by which exercise may offset hyperglycemic‐related oxidant damage (Paroo et al. 2002a,b).

Promising clinical data have shown that higher levels of exercise intensity, either aerobic or resistance exercise, may alleviate the onset of hypoglycemia in T1DM subjects through increased secretion of glucose counterregulatory defenses (Yardley et al. 2012, 2013; Armstrong and Sigal 2013). However, few studies have examined the cardiovascular benefits of these forms of exercise. In fact, many practitioners encourage patients with diabetes to avoid intense exercise, as vigorous physical activity can acutely and transiently increase the risk of a cardiovascular event (Thompson et al. 2007). However, a recent study in patients with coronary heart disease reported that although a low risk of experiencing a cardiovascular event is present, given the tremendous advantages over lower intensity exercise, high‐intensity exercise should be considered in a coronary heart disease rehabilitation setting (Rognmo et al. 2012). Indeed, our laboratory has shown that higher intensities of regular aerobic exercise in rats with T1DM leads to improvements in vasorelaxation responsiveness, insulin dose requirements and insulin sensitivity measures in comparison to lower intensities of exercise (Hall et al. 2013; Murias et al. 2013). Experimental work is needed to further understand the cardiovascular benefits associated with different modalities of exercise while taking into consideration which of these exercise modalities provide the lowest risk of exercise‐mediated hypoglycemia.

The present study employed an insulin‐treated STZ model of T1DM in which rats were maintained at moderately elevated blood glucose concentrations, which more accurately reflects the exercising patient population of T1DM. Using this model, we examined which mode of exercise training elicited the greatest cardiovascular benefit, as determined by recovery from ischemia‐reperfusion (I/R) injury. Secondly, we measured the blood glucose concentration of rats with T1DM prior to, and following exercise, in order to examine which mode of regular exercise delivers the lowest risk of hypoglycemia development. It was hypothesized that higher intensities of regular exercise such as resistance and high‐intensity aerobic exercise would lead to the greatest cardiovascular benefit while limiting the onset of exercise‐mediated hypoglycemia during the course of the exercise program.

## Research design and methods

Eight‐week‐old male Sprague–Dawley rats were obtained from Charles River Laboratories, housed two per cage in standard cages and maintained on a 12‐h dark/light cycle at a constant temperature (20 ± 1°C) and relative humidity (50%). Rats were allowed access to standard rat chow (Prolab‐RMH‐3000; PMI Nutrition International: 22% crude protein, 5% crude fat, 5% fiber, 6% ash), and water ad libitum. Ethics approval for the participation of rats in this study was acquired through the Research Ethics Board of the University of Western Ontario, which is in accordance with the guidelines of the Canadian Council on Animal Care.

### Experimental groups

Fifty rats were randomly assigned to one of five groups: (1) Nondiabetic sedentary control (C; *n* = 10), (2) diabetic sedentary control (CD; *n* = 10), (3) diabetic resistance exercise (DR; *n* = 10), (4) diabetic low‐intensity exercise (DL; *n* = 10), and (5) diabetic high‐intensity exercise (DH; *n* = 10).

### Diabetes Induction

Upon arrival rats were housed for a minimum of 5 days to allow them to become familiar with their new surroundings. T1DM was induced by administering 20 mg/kg of filtered (0.2 *μ*m) streptozotocin (STZ; Sigma Alderich, Oakville, ON, Canada) dissolved in a citrate buffer (0.1 mol/L, pH 4.5) via intraperitoneal (IP) injection on five consecutive days. Diabetes was confirmed by measuring two consecutive blood glucose concentrations of greater than 18 mmol/L. If diabetes confirmation was not obtained following five injections, the animals were given subsequent 20 mg/kg STZ‐IP injections until two readings of 18 mmol/L were obtained. Following the confirmation of diabetes, insulin pellets (LinShin, Toronto, ON, Canada) were implanted subcutaneously above the abdomen. According to manufactures instruction, each insulin pellet slowly releases insulin at an approximate rate of two International Units (IU) per day. Insulin pellet dosages were then monitored for 1 week and adjusted in order to obtain daily nonfasting blood glucose concentrations in the range of 9–15 mmol/L.

### Exercise protocols

The exercise protocols consisted of six weeks of regular exercise (5 days per week, starting at 9 am). DR rats were required to climb a ladder with a weighted bag secured to the proximal portion of their tail. The ladder was 1.1 m tall on an 80 degree incline with 2 cm spacing between rungs. This protocol describes an animal model of resistance exercise that closely resembles the exercise parameters and physiological adaptations observed in humans who participate in resistance training (Hornberger and Farrar 2004). During the first week of the exercise intervention, DR rats were familiarized to the weighted ladder climb. To do so, rats performed 10 climbs per day with varying weights attached to their tails (5%, 15%, 20%, and 35% of each rat's body mass). Between each climb, rats were allowed to rest for 120 sec in a 20 cm^3^ darkened box placed at the top of the ladder. The rest of the resistance training intervention (week two to six) consisted of rats carrying 50%, 75%, 90%, and 100% of their maximal lifting capacity for the first four climbs. Subsequent climbs were performed at 100% of their maximal lifting capacity until rats reached exhaustion (unable to finish a climb despite tactile stimulation to haunches). To determine maximal lifting capacity, rats were required to climb carrying 75% of their body weight on the initial climb. Thirty grams of weight was then added to each subsequent climb until rats reached exhaustion. The weight carried prior to exhaustion was marked as the new maximal lifting capacity. Maximal lifting capacity was determined and checked every four exercise sessions. During the first week of exercise, DL rats were familiarized on the motor‐driven treadmill (6 degree slope) at progressive running speeds of 7 m/min for 10 min, 11 m/min for 10 min, 13 m/min for 30 min, and 15 m/min for 10 min. Following familiarization (Weeks 2–6), DL rats exercised for 1 h per day at a speed of 15 m/min (6 degree slope). Week one of DH familiarization consisted of progressive running on the motor‐driven treadmill (6 degree slope) at 7 m/min for 10 min, 15 m/min for 10 min, 21 m/min for 30 min, and 24 m/min for 10 min. Following familiarization (Weeks 2–6), DH rats ran for 1 h per day on a 6 degree slope at a speed of 27 m/min. The exercise intensity chosen for DL and DH was approximately 50–60% and 70–80% of VO_2max_, respectively (Bedford et al. 1979). Continuous running during the aerobic exercise sessions was encouraged by small blasts of compressed air when rats broke a photoelectric beam close to the rear of the treadmill belt.

### Body weights and blood glucose concentrations

Body weights and blood glucose concentrations for C, CD, DR, DL, and DH were measured prior to STZ administration and exercise intervention, as well as prior to the last exercise session. Post‐exercise blood glucose measures were collected every 15 min for 2 h following an exercise bout during week three and week six of the exercise intervention. Weeks three and six were selected as the early and late phases of the exercise intervention, respectively. To remove any stress‐related changes in the blood glucose responses to exercise, week three was selected as the early time phase, as animals had become familiar with the mechanics of the exercise by this stage and require little encouragement to complete the 1 h exercise session. Blood was obtained from the saphenous vein (50 µL drop) and blood glucose concentrations were detected using a One Touch Ultra 2 Blood Glucose Monitoring System (Lifescan Canada Ltd, Burnaby, BC, Canada) and One Touch test strips (Lifescan Canada Ltd). Following blood collection at sacrifice, samples were clotted at room temperature for at least 30 min and then centrifuged at 10,000 rpm for 15 min at 4°C. Aliquots of serum were collected and analyzed for HDL cholesterol, total cholesterol, and triglycerides using a Hitachi 911 analyzer. Exogenous insulin concentrations were measured by ELISA (Alpco, Salem, NH: Catalog #80‐INSRT‐E01) following the exercise intervention.

### Heart collection and Langendorff preparation

Eighteen hours after their last exercise bout animals were anaesthetized via intraperitoneal injection of sodium pentobarbital (65 mg/kg) and sacrificed. Hearts were extracted and immediately arrested by placing them in ice‐cold Krebs–Henseleit buffer (KHB). Hearts were cannulated for unpaced retrograde aortic constant flow perfusion (15 mL/min) of coronary arteries with KHB (120 mmol/L NaCl, 4.63 mmol/L KCl, 1.17 mmol/L KH_2_PO_4_, 1.25 mmol/L CaCl_2_, 1.2 mmol/L MgCl_2_, 20 mmol/L NaHCO_3_, and 8 mmol/L glucose gassed with 95% O_2_, and 5% CO_2)_ maintained at 37°C (Paroo et al. 2002a). Hearts were equilibrated for 30 min to determine baseline function (preischemic value) and then flow was terminated for 50 min to induce ischemia. Following ischemia, hearts were subsequently reperfused for 30 min at 15 mL/min. Measures of left ventricular mechanical function were measured throughout the 30 min reperfusion period. These measures of left mechanical function include left ventricular developed pressure (LVDP), left ventricular end‐diastolic pressure (LVEDP), maximal rate of contraction (+dP/dt), and maximal rate of relaxation (−dP/dt). Each cardiac functional measure obtained during the reperfusion period was then converted to a percentage of preischemic values. Immediately following the ischemia‐reperfusion protocol, hearts were removed from the cannula and the left ventricle was dissected and frozen in liquid nitrogen and stored at −70°C until analyzed.

### Western blotting

Left ventricles were homogenized in a 1:10 (weight:volume) ratio of buffer (100 mmol/L NaCl, 50 mmol/L Tris base, 0.1 mmol/L EDTA, 0.1 mmol/L EGTA, and 1% Triton‐X 100, adjusted to pH ~7.5, 1% phosphatase inhibitor and 1% protease inhibitor) using a polytron, and centrifuged at 20,000 *g* for 20 min. The Bradford protein assay was used to calculate total protein concentration in each homogenate sample. Polyacrylamide gels were composed of a 10% acrylamide separating gel and 4% acrylamide stacking gel. Homogenates were placed in a 1:1 ratio of sample buffer (0.5 mol/L Tris base, 13% glycerol, 0.05% SDS, 13% 2‐beta‐mercaptoethanol, and bromophenol blue) to homogenates. Each gel contained a molecular weight marker to determine the molecular weight of the proteins. Electrophoresis was performed and proteins were run at a constant voltage of 150V for 1.5 h in running buffer (25 mmol/L Tris base, 200 mmol/L glycine, and 0.1% SDS, pH ~8.3). Gels were placed in transfer buffer (10% running buffer, 20% methanol, 70% ddH20) and proteins were electrophoretically transferred to nitrocellulose membranes at 70V for 1.5 h. The membranes were blocked in 5% nonfat dry milk (BioRad, Mississauga, ON 170‐6404) in Tris‐buffered saline (TTBS; 10 mmol/L Tris, 100 mmol/L NaCl, 0.1% Tween‐20, pH 7.5) for 1 h. Membranes were then incubated overnight at 4°C with primary antibodies specific to Hsp70 (Enzo Life Sciences Inc., Farmingdale, NY; 1:4000), MnSOD (Enzo Life Sciences Inc.; 1:2000), and Cu/Zn SOD (Enzo Life Sciences Inc.; 1:2000) in TTBS with 2% nonfat dry milk. After primary antibody incubation, nitrocellulose membranes were washed in TTBS 3 times for 10 min. Membranes were then incubated for 1 h with horseradish peroxidase‐conjugated secondary antibody specific for rabbit IgG (goat anti‐rabbit IgG‐HRP conjugate, BioRad, Mississauga, ON; 1:5000) diluted in TTBS with 2% nonfat dry milk. Membranes were again washed in TTBS 3 times for 10 min and subsequently imaged using chemiluminescent detection. A luminol‐based chemiluminescent substrate (BioRad, Mississauga, ON; Western C Enhanced Chemiluminescent Kit, 170‐5070) was placed on the membranes, allowing protein bands to become visible. Protein bands were captured using a BioRad Chemidoc XRS imager and optical densities were quantified using BioRad Quantity One software. Optical densities were normalized to a soleus‐positive control as well as *β*‐actin.

### Data analysis

Body weight, blood lipids, exogenous insulin, glucose concentrations, Hsp70, MnSOD, and Cu/ZnSOD protein content were compared via one‐way ANOVA. Post‐exercise blood glucose concentrations and Langendorff measures were compared using a two‐way repeated measures analysis of variance (ANOVA) test. When a significant interaction effect was found, a least squares difference post hoc test was performed. A significance level was set at *P* < 0.05. All data were expressed as mean ± SE. All statistical analyses were performed using SigmaPlot and SigmaStat computer software (Systat Software, Inc., San Jose, CA).

## Results

### Animal characteristics

In comparison to C, all diabetic animals (CD, DR, DL, and DH) demonstrated significantly lower mean body weight, while demonstrating significantly higher blood glucose concentrations prior to both the onset of the exercise intervention and the last bout of exercise (*P* < 0.05; Table 1). These measures did not differ across the diabetic groups (*P* > 0.05). Regular exercise reduced exogenous insulin requirements independent of the exercise modality (*P* < 0.05; DR, DL, and DH). Several blood lipids were significantly altered as a result of diabetes in comparison to nondiabetic animals (*P* < 0.05; Table 1). Triglyceride and LDL Cholesterol were significantly lower than C animals (*P* < 0.05), and were not altered as a result of the exercise intervention (*P* > 0.05). Total Cholesterol, Total Cholesterol:HDL were significantly lower in the exercise trained (DR, DL, and DH) animals and lowest in the DL animals (*P* < 0.05).

**Table 1.** General animal characteristics

	C	CD	DR	DL	DH
Blood glucose (mmol/L)					
Pre‐STZ administration	5.6 ± 0.09	5.8 ± 0.2	5.8 ± 0.2	5.3 ± 0.2	5.4 ± 0.2
Pre‐exercise intervention	5.6 ± 0.1	11.8 ± 1.2^1^	14.5 ± 1.5^1^	12.0 ± 1.7^1^	15.2 ± 0.5^1^
Prior to the last bout of exercise	5.13 ± 0.15	15.45 ± 0.84^1^	15.27 ± 0.75^1^	13.2 ± 1.24^1^	14.81 ± 0.76^1^
Body weight (g)					
Pre‐STZ administration	366 ± 7.88	371 ± 9.09	365 ± 6.94	345 ± 7.10	360 ± 8.10
Pre‐exercise intervention	408 ± 7.90	376 ± 5.81^1^	372 ± 5.34^1^	369 ± 7.33^1^	365 ± 8.48^1^
Prior to the last bout of exercise	574 ± 9.05	471 ± 9.05^1^	452 ± 11.16^1^	448 ± 21.72^1^	438 ± 8.43^1^
Exogenous insulin (IU)		25.41 ± 10.58	11.90 ± 3.62^2^	14.53 ± 7.46^2^	11.00 ± 3.77^2^
Total cholesterol (mmol/L)	1.81 ± 0.13	1.63 ± 0.11	1.42 ± 0.07^1^	1.33 ± 0.07^1^^,^^2^	1.59 ± 0.03
Triglycerides (mmol/L)	1.72 ± 0.13	1.09 ± 0.14^1^	1.16 ± 0.20^1^	0.857 ± 0.18^1^	1.09 ± 0.13^1^
HDL cholesterol (mmol/L)	0.78 ± 0.16	1.11 ± 0.15	0.10 ± 0.08	0.98 ± 0.06	1.03 ± 0.03
Total cholesterol:HDL	1.90 ± 0.3	1.67 ± 0.09	1.59 ± 0.13^1^	1.38 ± 0.05^1^^,^^2^	1.54 ± 0.04^1^
LDL cholesterol (mmol/L)	1.95 ± 0.30	0.84 ± 0.21^1^	0.84 ± 0.32^1^	0.45 ± 0.15^1^	0.77 ± 0.18^1^

Data are means ± SE; *n* = 10.

^1^ Significantly different from C (*P* < 0.05).

^2^ Significantly different from CD (*P* < 0.05).

### Left ventricle mechanical performance

**Figure 1.**
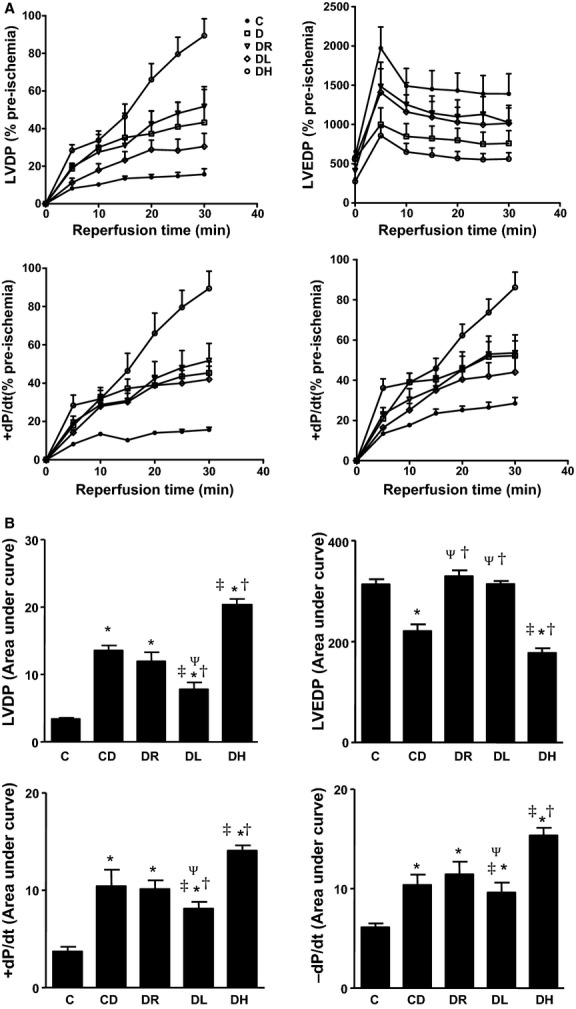
Effect of T1DM and exercise training modality on left ventricle mechanical function. The data are presented in time course format (A) and area under the curve measurement (B). Compared with control and sedentary diabetic high intensity aerobic exercise training lead to enhancement in postischemic LVDP, +dP/dt, −dP/dt and LVEDP in rat hearts following ischemic stress. *different than C; ^†^different than CD; ^‡^different than DR; ^Ψ^different than DH; *P* < 0.05, based on a one‐way ANOVA. Data presented as a mean ± SE.

Compared to C, diabetic groups (CD, DL, DR, and DH) demonstrated greater cardiac functional performance in postischemic left ventricular developed pressure (LVDP), maximal rate of contraction (+dP/dt), and maximal rate of relaxation (‐dP/dt) (Fig. 1; *P *<**0.05). The demonstrated protective effects of exercise, as assessed by left ventricular performance, were not consistent across each exercise modality. DH animals showed the greatest positive benefit in cardiovascular recovery in comparison to CD and DR in all four variables examined (LVDP, LVEDP, +dP/dt, −dP/dt) (*P *<**0.05). Both the DH and CD animals showed significantly lower left ventricular end‐diastolic pressure (LVEDP), which was not significantly different amongst the rest of the groups (*P* < 0.05).

### SDS‐PAGE and Western blot analysis

**Figure 2.**
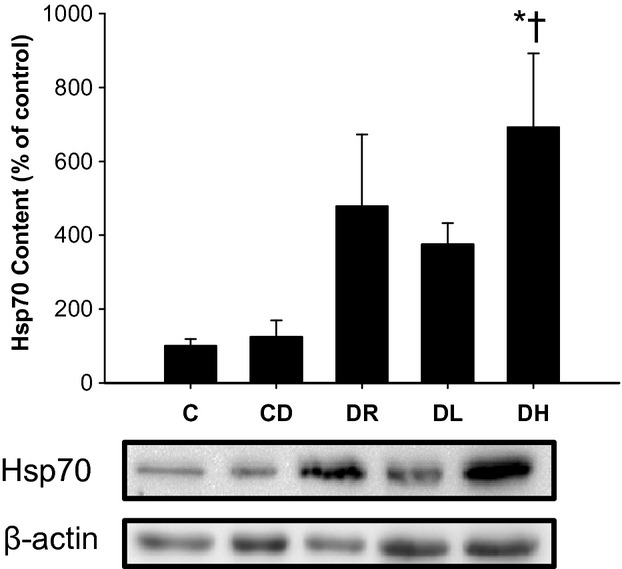
Effect of T1DM and exercise training modality on left ventricle Hsp70 protein content. High‐intensity aerobic exercise training led to enhancement in Hsp70 expression in rat hearts compared to sedentary rats. *different than C; ^†^different than CD,* P* < 0.05, based on a one‐way ANOVA. Data presented as a mean ± SE.

**Figure 3.**
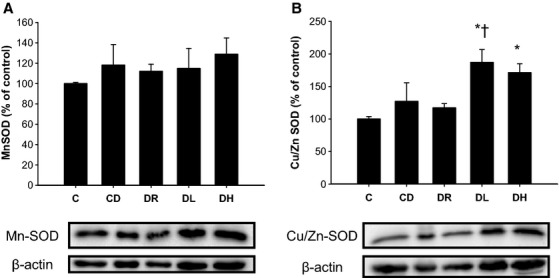
Effect of diabetic stress and training on left ventricle MnSOD (A) and Cu/Zn SOD (B) protein content. No change in MnSOD was evident across experimental groups while both low‐ and high‐intensity exercise aerobic training led to elevations in Cu/Zn SOD when compared to control rats. Low‐intensity aerobic exercise also demonstrated significantly more Cu/Zn SOD compared to sedentary diabetic rats *different than C; ^†^different than CD,* P* < 0.05, based on a one‐way ANOVA. Data presented as a mean ± SE.

Compared to both C and CD, DH rats exhibited higher cardiac Hsp70 expression (Fig. 2; *P* < 0.05). Hsp70 content of both DR and DL trained rats were not significantly different from either C or CD (*P* > 0.05). There were no differences in MnSOD across experimental groups (Fig. 3A; *P* > 0.05). Compared to C, both DL and DH had significantly higher expression levels of Cu/Zn SOD, while DL had significantly elevated levels of Cu/Zn SOD in comparison to CD (Fig. 3B; *P* < 0.05).

### Exercise‐mediated blood glucose reductions

**Figure 4.**
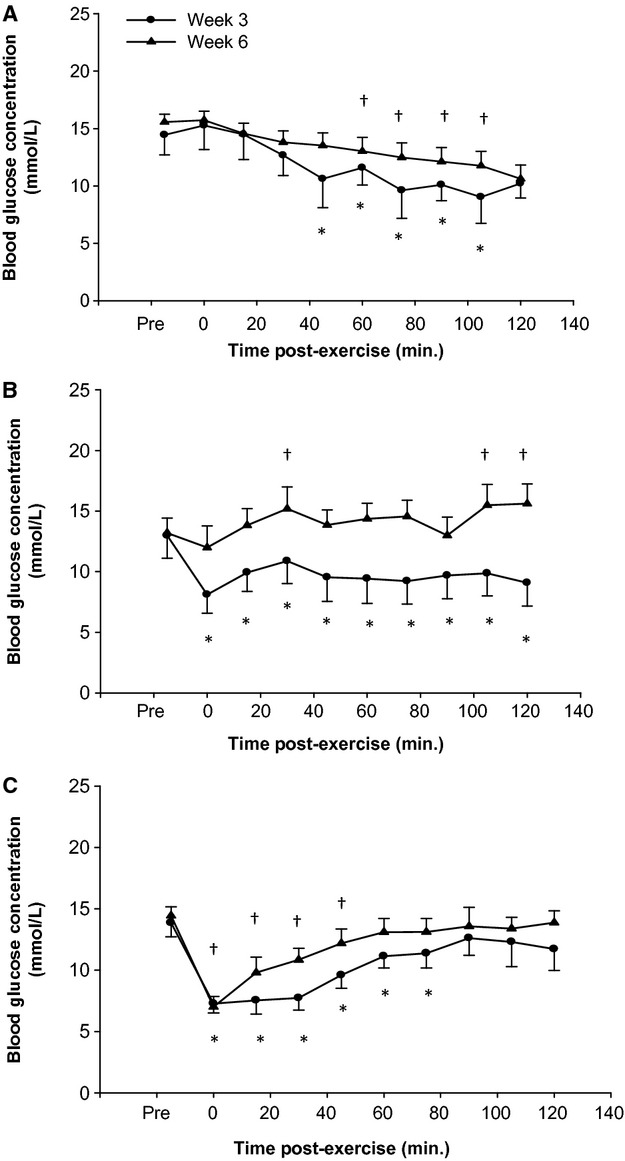
Two hour post‐exercise blood glucose measures. Blood glucose was measured in (A) DR (B) DL and (C) DH every 15 min for 2 h post‐exercise. DR and DH demonstrated no significant difference in post‐exercise blood glucose response at week 3 and week 6; DL demonstrated an attenuated blood glucose response at week 6 compared to week 3 (*P* < 0.05). An asterisk (*) indicates a significant difference in blood glucose from pre‐exercise (PRE) at week 3 of exercise training based on the post hoc test (*P* < 0.05). A cross (†) indicates a significant difference in blood glucose from pre‐exercise (PRE) at week 6 of exercise training based on the post hoc test (*P* < 0.05). Data presented as a mean ± SE.

At week three, blood glucose concentrations in DR animals declined slowly following the exercise, reaching significantly lower concentrations (vs. pre‐exercise) at 45 min post‐exercise, and remained significantly lower until 110 min post‐exercise (Fig. 4A; *P* < 0.05). In DL and DH animals, the decline in blood glucose concentrations in response to exercise during week three was evident immediately following the exercise session (Fig. 4B and C; *P* < 0.05). The reduction in blood glucose concentrations of DL rats remained significantly lower (vs. pre‐exercise) at 120 min post‐exercise, while DH rats returned to pre‐exercise blood glucose concentrations by 90 min post‐exercise (*P* < 0.05). At week six of training, DR rats demonstrated a delayed blood glucose reduction in response to exercise in comparison to the week three exercise session, whereby a significant drop in blood glucose was not evident until 60 min post‐exercise (*P* < 0.05). Similarly, DH rats demonstrated an altered blood glucose response to exercise at week six, exhibiting a quicker return to pre‐exercise blood glucose concentrations by 60 min (*P* < 0.05). At week six, a drop in blood glucose was absent in DL rats, but rather an increase in blood glucose was evident at 30, 105, and 120 min post‐exercise (*P* < 0.05).

Despite a reduction in blood glucose concentrations being apparent across the different modalities of exercise, the extent of the drop in blood glucose did not approach hypoglycemic concentrations (<3.0 mmol/L) in either week three or six the exercise intervention.

## Discussion

Using an insulin‐treated model of T1DM, the purpose of the present study was to investigate the cardiovascular benefit of several different exercise regimes which have been previously shown clinically to minimize the onset of hypoglycemia evoked by exercise (Marliss and Vranic 2002; Yardley et al. 2013). Here, we demonstrate that the extent of exercise‐related protection from ischemia‐reperfusion (I/R) damage appears to be dependent on the exercise modality, whereby high‐intensity aerobic exercise demonstrates the greatest recovery following an ischemic insult. Secondly, although insulin‐treated rats with T1DM demonstrated a significant drop in blood glucose regardless of exercise modality, blood glucose concentrations failed to reach hypoglycemic concentrations (<3.0 mmol/L) and aerobic exercise was associated with the fastest return to pre‐exercise glucose concentrations. These findings would suggest that each exercise modality can be performed safely, at least under conditions in which blood glucose concentrations are elevated before exercise is initiated; however, the greatest cardiovascular benefit is evident following high‐intensity aerobic exercise.

In partial agreement with our hypothesis, these results demonstrate an intensity‐related effect on the reduction in I/R‐injury in aerobically trained rats. These findings would support previous literature in otherwise healthy individuals in which exercise performed at higher relative intensities elicited greater cardiovascular benefit than moderate intensity levels of exercise, independent of the volume of activity (Lee et al. 2003). Less information is available with regards to the impact of higher levels of exercise intensity in patients with T1DM, a population well characterized to be at greater risk for cardiovascular disease (Orchard et al. 2006). In line with these findings, our laboratory has previously reported that high‐intensity aerobic exercise training is able to restore the diabetes‐related loss of nerve arterial vasodilation, decreased vascular responsiveness, and insulin sensitivity (Hall et al. 2013; Murias et al. 2013; Olver et al. 2014). In contrast to high‐intensity aerobic training, we found little evidence of cardiovascular protection from I/R‐injury following six weeks of resistance exercise (DR) and low‐intensity aerobic exercise (DL) in comparison to sedentary diabetic (CD) rats. To our knowledge, the present investigation is one of the first studies to have measured I/R‐injury in resistance exercised animals with T1DM. Doustar et al. (2012) examined the impact of a 4‐week resistance training program on I/R‐injury in healthy rats and reported no exercise‐related benefits. However, in a subsequent study it was demonstrated that 12 weeks of resistance training led to significant improvements in coronary flow, developed pressure, diastolic pressure, and infarct size, suggesting that this form of exercise can be effective for cardiac protection (Soufi et al. 2011). Thus, the current findings may be reflective of the slower beneficial adaptations this form of exercise training may have in comparison to aerobic exercise. Indeed, endurance exercise has been shown to elicit rapid cardiovascular adaptations following as little as 6 days of training (Green et al. 1991; Goodman et al. 2005). While it is difficult to extrapolate these reports to our model of T1DM, it is plausible that lengthening the resistance training program may lead to enhanced cardiac protection to a similar degree as high‐intensity exercise.

Our laboratory, as well as others, have demonstrated that exercise‐related increases in Hsp70 can protect the myocardium against I/R‐injury (Hutter et al. 1996; Benjamin and McMillan 1998; Paroo et al. 2002a; Melling et al. 2007). It has been established that Hsp70 protein is reduced in several tissues including the heart in severely hyperglycemic rats with STZ‐induced T1DM (Atalay et al. 2004). Further, the elevated expression of Hsp70 in the heart which normally occurs following moderate intensity aerobic exercise is suppressed in untreated STZ‐induced T1DM animals (Atalay et al. 2004). Using the same insulin‐treated diabetic model used in the current study, we recently reported that rats with T1DM are able to elicit elevations in constitutive Hsp70 content in the heart to similar levels as non‐T1DM controls following moderate intensity exercise training (Melling et al. 2013). These discrepancies are likely due to the use of insulin supplementation in our rat model of T1DM. It has been established that insulin treatment alone can increase Hsp70 expression, as well as enhance myocardial recovery of contractile function postischemic injury (Li et al. 2006). Here, we demonstrate that the elevations in heart Hsp70 expression were modality specific, and positively associated with the level of cardiovascular protection. High‐intensity regular aerobic exercise (DH) demonstrated a significant elevation in Hsp70 and exhibited the largest recovery from I/R‐injury. In contrast, low‐intensity regular aerobic exercise (DL) did not exhibit evidence of improved cardioprotection nor did this exercise regime display significant increases in heart Hsp70. Indeed, it has been reported that an intensity‐dependent threshold exists in the expression of Hsp70 following aerobic exercise in otherwise healthy animals (Milne and Noble 2002).

A notable finding of the current study is that in comparison to C animals, CD animals demonstrated some level of protection from I/R‐injury. This ischemia resistance has been demonstrated in STZ models of T1DM elsewhere in the literature (Tani and Neely 1988; Khandoudi et al. 1990) and may be due to the duration and severity of the diabetic state (for detailed review, see (Paulson 1997)). Enhanced antioxidant defenses may contribute to this heightened cardioprotection, as it has been reported that animals with STZ‐induced T1DM demonstrate elevations in antioxidant defenses, such as catalase, Mn‐SOD, and Glutathione S Transferase (GST) levels, which are evident as early as two weeks following STZ administration (Thompson et al. 1992; Ivanović‐Matić et al. 2010). Although rats with T1DM in the present study did not exhibit changes in heart MnSOD or Cu/Zn SOD protein, it is possible that their activity or other antioxidant defenses may be elevated and contribute to increased cardioprotection (Ravingerová et al. 2010). It has been shown that hearts of rats with T1DM demonstrate a decrease in the accumulation of glycolytic products during ischemia (lactate and protons), which has been proposed to be beneficial during an ischemic event (Feuvray and Lopaschuk 1997). Further, heart of T1DM animals have been shown to have alterations in intracellular calcium signaling, which has been shown to induce protection in the normal heart (Ravingerová et al. 2010). It is important to note that enhanced protection from I/R‐injury has also been reported in patients with T1DM undergoing forearm ischemia (Engbersen et al. 2012). Increased nitric oxide production coupled with changes in vascular diameter may also contribute to improved cardiovascular function in the early stages of STZ‐induced diabetes (Van Dam et al. 2000). Lastly, rats with T1DM in the current study were supplemented with insulin, which may have put them in a heightened protective state in comparison to control animals (Li et al. 2006). Nonetheless, the current data provide evidence that aerobic exercise at higher levels of intensity can greatly improve cardiovascular function in insulin‐treated rats with T1DM. Further work is still required to fully understand the susceptibility of patients with T1DM to damage during ischemic stress and the impact of insulin treatment on the severity of the stress.

While benefits in cardiovascular function and protection from I/R‐damage are evident following high‐intensity regular aerobic exercise, a primary consideration when prescribing exercise to populations with T1DM is the elevated risk of experiencing hypoglycemia. Exercise‐mediated reductions in blood glucose concentrations differed based on the exercise modality, an observation well documented in patient populations with T1DM (Iscoe and Riddell 2011; Davey et al. 2013; Yardley et al. 2013). In the present investigation, we observed that at week three of the exercise intervention both aerobic exercise modalities (low and high intensity) led to a significant drop in blood glucose immediately post‐exercise. In contrast, resistance exercise resulted in a more gradual decline in blood glucose, which did not return to pre‐exercise values until approximately 4 h post‐exercise (data from 2–4 h not shown). This gradual decline in blood glucose in response to resistance exercise may be more beneficial for patients with T1DM, as it provides ample time to combat the drop in blood glucose following exercise. Precautionary measures such as carbohydrate ingestion or a decrease in insulin dosage could mitigate the reduction in blood glucose all together. Managing the immediate drop in blood glucose which occurs in response to aerobic exercise is more challenging, and as such, patients often compensate by elevating blood glucose concentrations prior to exercise through carbohydrate ingestion or insulin dosage adjustments (Tsalikian et al. 2006; Younk et al. 2011). However, it is important to note that despite the rapid drop in blood glucose, exercising rats with T1DM failed to reach hypoglycemic blood glucose concentrations (<3.0 mmol/L) when they started with moderately elevated blood glucose concentrations. As such, maintaining blood glucose concentrations at a higher target range prior to exercise, significantly reduces the risk of hypoglycemia associated with aerobic exercise.

In a recent publication, our group demonstrated that the immediate exercise‐induced reduction in blood glucose in response to moderate intensity aerobic exercise is unaltered after ten weeks of training (McDonald et al. 2014). Here, it appears that the magnitude of blood glucose reduction in response to different exercise modalities, and the return to pre‐exercise blood glucose concentrations may adapt during the course of the six‐week exercise intervention. For instance, an elevation in blood glucose was evident post‐exercise following six weeks of low‐intensity regular aerobic exercise rather than a significant decline in blood glucose, which was apparent at week three. This is of particular interest, given that insulin sensitivity is heightened following exercise training and it is unlikely that a robust sympathetic response would be present at this level of exercise intensity (Steppel and Horton 2003; Hall et al. 2013). However, it is likely that a reduction in the reliance on blood glucose as an energy source occurred, as running speed was consistent across the treatment period. Thus, the relative exercise intensity likely declined toward the later stages of the experiment, which in turn may have led to a shift in fuel source from primarily carbohydrate to fat (Brouns and van der Vusse 1998). On the other hand, high‐intensity aerobic and resistance exercised rats displayed a smaller blood glucose adaption post‐exercise following the exercise intervention. It is plausible that the higher level of intensity in these exercise modalities led to a similar, or even increased (resistance exercise) glucose demand during the course of the exercise intervention period.

In summary, we demonstrate that the extent of exercise‐related protection from I/R‐damage appears to be dependent on exercise modality, whereby higher intensities of aerobic exercise demonstrate the greatest recovery following an ischemic insult. Secondly, although the magnitude and time course are different, a significant decrease in blood glucose is apparent following each modality of exercise. Maintaining rats at a higher target blood glucose concentration prior to exercise reduced the risk of post‐exercise hypoglycemia. These findings would suggest that each modality of exercise can be performed safely if blood glucose levels are elevated prior to initiating exercise; however, the greatest benefits are evident following high‐intensity aerobic exercise.

## Acknowledgments

The authors thank Michelle Dotzert for contributing to data analysis.

## Conflict of Interest

None declared.
